# Research on Two-Stage Semi-Active ISD Suspension Based on Improved Fuzzy Neural Network PID Control

**DOI:** 10.3390/s23208388

**Published:** 2023-10-11

**Authors:** Linhao Jin, Jingjing Fan, Fu Du, Ming Zhan

**Affiliations:** 1 School of Electrical and Control Engineering, North China University of Technology, Beijing 100144, China; 2 School of Mechanical and Vehicular Engineering, Beijing Institute of Technology, Beijing 100081, China

**Keywords:** two-stage, ISD semi-active suspension, grey wolf optimization algorithm, fuzzy neural network, Matlab/Simulink

## Abstract

To better improve the ride comfort and handling stability of vehicles, a new two-stage ISD semi-active suspension structure is designed, which consists of the three elements, including an adjustable damper, spring, and inerter. Meanwhile, a new semi-active ISD suspension control strategy is proposed based on this structure. Firstly, the fuzzy neural network’s initial parameters are optimized using the grey wolf optimization algorithm. Then, the fuzzy neural network with the optimal parameters is adjusted to the PID parameters. Finally, a 1/4 2-degree-of-freedom ISD semi-active suspension model is constructed in Matlab/Simulink, and the dynamics simulation is carried out for the three schemes using PID control, fuzzy neural network PID control, and improved fuzzy neural network PID control, respectively. The results show that compared with adopting PID control and fuzzy neural network PID control strategy, the vehicle body acceleration and tire dynamic loads are significantly reduced after using the grey wolf optimized fuzzy neural network PID control strategy, which shows that the control strategy proposed in this paper can significantly improve the vehicle smoothness and the stability of the handling.

## 1. Introduction

A vehicle suspension system is a connecting and force-transmitting device between the body (or frame) and the wheels (axle) [1]. It can transfer the forces exerted by the road on the wheels to the vehicle body, and can play the role of cushioning and suppressing the vehicle body vibration. Therefore, the performance of the suspension will greatly affect the vehicle smoothness and the stability of the handling [2]. However, in the development of suspension so far, whether it is passive suspension, semi-active suspension, or active suspension, scholars for the suspension control method research endlessly, but the basic structure of the suspension is still composed of a “spring-damper”. To improve the vibration isolation performance of the suspension, some researchers carried out topology higher-order processing for the “spring-damper”, which can improve the suspension performance, but it is difficult to apply; so how to effectively improve the vibration isolation performance of the suspension became a bottleneck [3]. However, in 2002, Professor Smith of Cambridge University proposed the concept of inerter [4], which completely broke the basic structure of the “spring-damper”. As a kind of two-endpoint element, the inertia force generated by the inerter is proportional to the relative acceleration of the two endpoints, so it can be used in a vibration isolation system [5].

A large number of researchers and scholars gradually applied the inerter to the automobile suspension, so suspension systems composed of an inerter, spring, and damper came into being. From an industrial perspective, the manufacturing cost of this suspension is relatively low. The components are relatively simple, making them easier to maintain and repair. This makes it more durable in certain harsh conditions. After a large number of experiments, it was proved that this suspension can greatly improve the vibration performance of the vehicle suspension [6,7]. At the same time, domestic and foreign researchers conducted a large number of studies on the ISD suspension structure and the optimization of suspension parameters [8,9,10]. Zhang Xiaoliang et al. studied the ISD vehicle suspension and proved that the introduction of inerter can improve the vibration isolation performance [11,12]. Kuznetsov et al. established a 1/4 ISD vehicle suspension model and analyzed the effect of different road parameters on ride comfort [13]. Yang et al. designed an ISD suspension containing a power absorber structure and analyzed the vibration damping performance of ISD suspension in the frequency domain perspective [14].

For ISD suspension control, Yinlong Hu investigated the ISD suspension based on the skyhook inerter strategy, which enhanced the ride comfort through anti-shock and tremor, switching control and continuous control modes [15]. Xinjie Zhang [16] compared the performance of the semi−active ISD suspension using skyhook, groundhook, and hybrid skyhook control, respectively, and the results show that the semi−active suspension with the hybrid skyhook strategy has better performance in ride comfort and handling stability. Wang Ruochen used a fuzzy control strategy for ISD semi−active suspension, and the results show that the semi-active ISD suspension had better vibration damping performance compared to the conventional semi-active suspension [17].

Among various control methods, PID control stands out in the field of vehicle suspension due to its simple control principles, easy implementation, fast computation, adaptability to a wide range of different systems, robustness, and cost-effectiveness. It is widely applied in vehicle suspension systems [18]. Despite the emergence of various advanced control algorithms in the suspension field in recent years, such as fuzzy control and neural network control, PID control remains the most widely used method in engineering. Therefore, many researchers continue to study PID control strategies. However, traditional PID controllers have difficulty in accurately setting parameters and remain fixed once configured. As vehicle conditions change, PID controllers may not adapt to all suspension scenarios [19]. To improve the control effectiveness of PID controllers in vehicle suspension, some scholars introduced fuzzy control into semi−active suspension control. Fuzzy systems are good at expressing structural and vague knowledge, making them suitable for addressing nonlinearity, uncertainty, and other complex issues [20]. For instance, Wang Lin et al. [21] designed a fuzzy PID controller with spring displacement and its rate of change as fuzzy control inputs and the three tuning parameters of PID as outputs. They demonstrated that this control method can effectively reduce vehicle body acceleration and suspension deflection. Zeng et al. [22] used a genetic algorithm to optimize the quantization factors of the fuzzy controller and the proportional factors of the PID tuning formula. They designed a semi-active suspension fuzzy PID controller that dynamically adjusts PID parameters through fuzzy inference in real time, effectively enhancing vehicle performance.

While fuzzy control relies solely on empirical knowledge and is not suitable for situations requiring multivariable control, neural network control excels in its strong self-learning abilities and applicability in complex environments with multiple inputs and outputs. However, it is less adept at describing control rules [23]. Therefore, the emergence of fuzzy neural networks was a natural progression. They combine the advantages of neural networks and fuzzy systems. From a control perspective, their strengths include robust control systems. The design of fuzzy neural networks is based on the knowledge and expertise of domain experts, allowing for the construction of fuzzy rules tailored to specific domain requirements. This means that designers can directly incorporate domain-specific knowledge to enhance performance or adapt to particular tasks. Fuzzy neural networks can be manually adjusted and modified to meet the needs of specific domains, accommodating new situations and requirements. This flexibility of human intervention allows for rapid system adjustments when necessary [24]. Therefore, compared to reinforcement learning and other neural network methods, fuzzy neural networks are more widely applicable and useful in the research of vehicle suspension systems. Phu D X et al. designed a fuzzy neural network controller to be applied in a semi−active automotive seat suspension, and the results show that the designed controller had better vibration damping performance compared with the sliding mode controller [25]. Cheng Xinming et al., by designing the ANFIS controller and applying it in a semi−active suspension system, showed that the use of an ANFIS controller can reduce the vertical acceleration of the vehicle body and improve the smoothness of the vehicle [26]. Tian Fengfu et al. used 1/4 automobile suspension as a research object and used a fuzzy neural network to rectify the parameters of the PID controller, and the results show that the control strategy can improve the vehicle smoothness and ride comfort [27]. The learning process of fuzzy neural networks employs gradient descent, which is highly susceptible to becoming trapped in local optima. Therefore, some researchers introduced intelligent optimization algorithms to enhance the learning process by combining learning algorithms with intelligent optimization algorithms to achieve the best search results. Common intelligent optimization algorithms include particle swarm optimization, grey wolf optimization, and more. Lu et al. optimized BP neural networks for prediction using the grey wolf optimization algorithm, and the results demonstrate that compared to genetic algorithms and particle swarm optimization, the BP neural network with the grey wolf optimization algorithm achieved faster convergence and more favorable prediction performance [28]. Additionally, Fan et al. used the grey wolf optimization algorithm to optimize a fuzzy PID controller, providing evidence that the fuzzy PID controller optimized with grey wolf optimization outperformed the conventional fuzzy PID controller [29].

Recently, some scholars focused on the research of two−stage ISD suspension, Li Xiaopeng et al. established a single-wheel model of two−stage ISD suspension, and through simulation experiments, they concluded that the two-stage ISD suspension has a better low−frequency vibration damping performance than the classical ISD suspension [30]. Given that research on the two-stage ISD semi−active suspension system is currently in its infancy, and considering future engineering practical applications, in combination with the research findings of the aforementioned researchers, this paper proposes a grey wolf optimal control strategy based on fuzzy neural network PID control. The real-time control performance of the fuzzy neural network is utilized to achieve real-time tuning of the PID controller parameters. However, the fuzzy neural network has many parameters, so the grey wolf optimization method is first used to obtain a set of optimal parameters, which can avoid the gradient explosion caused when the neural network performs back propagation of errors. Finally, through MATLAB/Simulink simulation, it is demonstrated that the fuzzy neural network PID control strategy of grey wolf optimization has a better damping performance for ISD suspension.

The rest of the paper is organized as follows: Section 2 gives the 1/4 2 degrees of freedom two-stage ISD vehicle suspension system dynamics model and road excitation model, and then the principles of the fuzzy neural network PID control strategy and the grey wolf optimization algorithm and the optimization of the fuzzy neural network PID algorithm based on the grey wolf algorithm are presented, respectively. Section 3 gives the simulation in Matlab/Simulink and comparative analysis. Finally Section 4 concludes and summarizes the paper.

## 2. Materials and Methods

### 2.1. ISD Suspension Dynamics Model

**Figure 1:**
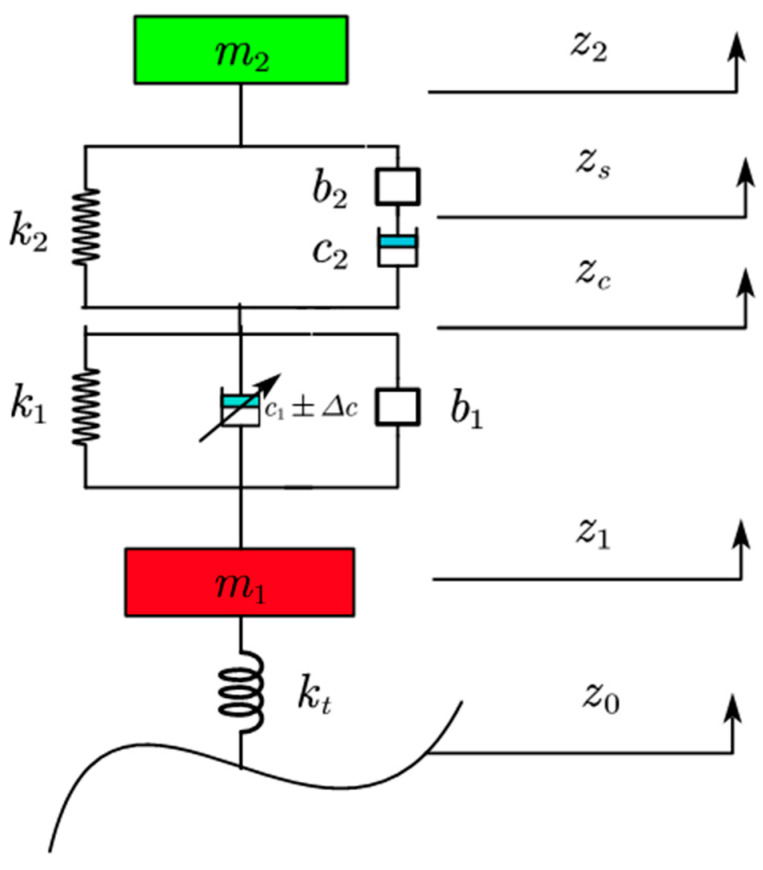
Two−stage ISD semi-active vehicle suspension model.

The two−stage ISD semi-active vehicle suspension model with 1/4 2 degrees of freedom is shown in Figure 1 below.

As shown in Figure 1, $m_{1}$ is the unsprung mass, $m_{2}$ is the sprung mass, $k_{t}$ is the tire stiffness, $k_{1}$ is the first stage stiffness coefficient, $k_{2}$ is the second stage stiffness coefficient, $c_{1}$ is the adjustable damper (its two ends can produce damping force), $c_{2}$ is the second stage damping coefficient, $b_{1}$ is the first stage inerter, $b_{2}$ is the second stage inerter, $z_{0}$ is the road excitation displacement, $z_{1}$ is the unsprung mass displacement, $z_{2}$ is the sprung mass displacement, and $z_{c}$ is the displacement between the common end of the suspension in suspension system.

Utilizing Newton’s second law, Figure 1 is written in the form of differential equations:

(1) $$\left\{ \begin{array}{l} m_{2}{\ddot{z}}_{2}=-k_{2}(z_{2}-z_{c})-u_{c}\\ m_{1}{\ddot{z}}_{1}+k_{t}(z_{1}-z_{0})-k_{1}(z_{c}-z_{1})-b_{1}({\ddot{z}}_{c}-{\ddot{z}}_{1})-(c_{1}\pm \Delta c)({\dot{z}}_{c}-{\dot{z}}_{1})=0\\ -k_{2}(z_{2}-z_{c})-u_{c}=-k_{1}(z_{c}-z_{1})-b_{1}({\ddot{z}}_{c}-{\ddot{z}}_{1})-(c_{1}\pm \Delta c)({\dot{z}}_{c}-{\dot{z}}_{1})\\ u_{c}=b_{2}({\ddot{z}}_{2}-{\ddot{z}}_{s})=c_{2}({\dot{z}}_{s}-{\dot{z}}_{c}) \end{array}\right.$$

where $c_{1}$ is an adjustable damper, its adjustment range is limited. Therefore, the adjustable damper can be equated to a constant value damper $c_{1}$ and a variable damper Δc. The damping force generated at both ends of the adjustable damper is shown in Equation (2) below.

(2) $$F_{c}=F_{c1}+F_{\Delta c}=c_{1}({\dot{z}}_{c}-{\dot{z}}_{1})+\Delta c({\dot{z}}_{c}-{\dot{z}}_{1})$$

Define the state variables as.

$$\left\{ \begin{array}{l} x_{1}=z_{2}\\ x_{2}={\dot{z}}_{2}\\ x_{3}=z_{1}\\ x_{4}={\dot{z}}_{1}\\ x_{5}=z_{c}\\ x_{6}={\dot{z}}_{c}\\ x_{7}=z_{s}\\ x_{8}={\dot{z}}_{s} \end{array}\right.$$

Then the state space expression for the differential equation of Equation (1) is given as:

(3) $$\left\{ \begin{array}{l} \dot{x}=Ax+BU\\ y=Cx+DU \end{array}\right.$$

where

$$\begin{array}{l} x={\left[\begin{array}{cccccccc} z_{2} & {\dot{z}}_{2} & z_{1} & {\dot{z}}_{1} & z_{c} & {\dot{z}}_{c} & z_{s} & {\dot{z}}_{s} \end{array}\right]}^{T} \end{array}$$

$$U={\left[\begin{array}{cc} F_{c} & z_{0} \end{array}\right]}^{T}$$

$$y={\left[\begin{array}{cccc} {\dot{z}}_{2} & {\ddot{z}}_{2} & k_{t}(z_{1}-z_{0}) & z_{2}-z_{1} \end{array}\right]}^{T}$$

$$A=\left[\begin{array}{cccccccc} 0 & 1 & 0 & 0 & 0 & 0 & 0 & 0\\ -\frac{k_{2}}{m_{2}} & 0 & 0 & 0 & \frac{k_{2}}{m_{2}} & \frac{c_{2}}{m_{2}} & 0 & -\frac{c_{2}}{m_{2}}\\ 0 & 0 & 0 & 1 & 0 & 0 & 0 & 0\\ \frac{k_{2}}{m_{1}} & 0 & -\frac{k_{t}}{m_{1}} & 0 & -\frac{k_{2}}{m_{1}} & -\frac{c_{2}}{m_{1}} & 0 & \frac{c_{2}}{m_{1}}\\ 0 & 0 & 0 & 0 & 0 & 1 & 0 & 0\\ \frac{k_{2}}{m_{1}}+\frac{k_{2}}{b_{1}} & 0 & \frac{k_{1}}{b_{1}}-\frac{k_{t}}{m_{1}} & \frac{c_{1}}{b_{1}} & \frac{-1-k_{1}}{b_{1}}-\frac{k_{2}}{m_{1}} & -\frac{c_{2}}{b_{1}}-\frac{c_{1}}{b_{1}}-\frac{c_{2}}{m_{1}} & 0 & \frac{c_{2}}{b_{1}}+\frac{c_{1}}{m_{1}}\\ 0 & 0 & 0 & 0 & 0 & 0 & 0 & 1\\ -\frac{k_{2}}{m_{2}} & 0 & 0 & 0 & \frac{1}{m_{2}} & \frac{c_{2}}{m_{2}}+\frac{c_{2}}{b_{2}} & 0 & -\frac{c_{2}}{m_{2}}-\frac{c_{2}}{b_{2}} \end{array}\right]$$

$$B=\left[\begin{array}{cc} 0 & 0\\ 0 & 0\\ 0 & 0\\ 0 & 0\\ 0 & 0\\ -\frac{1}{b_{1}} & \frac{1}{m_{1}}\\ 0 & 0\\ 0 & 0 \end{array}\right]$$

$$C=\left[\begin{array}{cccccccc} 0 & 1 & 0 & 0 & 0 & 0 & 0 & 0\\ -\frac{k_{2}}{m_{2}} & 0 & 0 & 0 & \frac{k_{2}}{m_{2}} & \frac{c_{2}}{m_{2}} & 0 & -\frac{c_{2}}{m_{2}}\\ 0 & 0 & k_{t} & 0 & 0 & 0 & 0 & 0\\ 1 & 0 & -1 & 0 & 0 & 0 & 0 & 0 \end{array}\right]$$

$$D=\left[\begin{array}{cc} 0 & 0\\ 0 & 0\\ 0 & -k_{t}\\ 0 & 0 \end{array}\right]$$

### 2.2. Road Excitation Model

#### 2.2.1. Stochastic Road Surface Model

It is very important to establish the road excitation model for the study of the vibration characteristics of the vehicle suspension system, which is usually described by the power spectral density of a road surface. According to ISO/TC108/SC2N67 and GB7031, the power spectrum density of a road is shown in the following equation [31]:

(4) $$G(n)=G(n_{0}){\left(\frac{n}{n_{0}}\right)}^{-\omega }$$

where n means the spatial frequency, $n_{0}$ is usually taken as 0.1, and ω means the frequency index is usually selected as 2.

When $n_{0}=0.1m^{-1}$, ω=2. According to the different values of the road unevenness coefficient, the road unevenness $G(n_{0})$ can be divided into many different grades: Grade A road surface simulates highway pavement, Grade B road surface simulates asphalt and concrete composite pavement, Grade C road surface simulates asphalt and gravel mixed pavement, and Grade D road surface simulates gravel pavement. This paper will focus on the discussion of Grade A–D road, so the geometric mean value of A–D-grade pavement is given, as shown in Table 1.

The road excitation model is usually described using first-order-filtered white noise with the time domain expression shown in Equation (5) below:

(5) $$\dot{x}(t)=-2\pi \cdot 0.1\cdot v\cdot x(t)+2\pi n_{0}\sqrt{G(n_{0})v}\cdot \omega (t)$$

#### 2.2.2. Sinusoidal Road Surface Model

The sinusoidal road surface is a commonly used road excitation model for studying suspension vibration characteristics. It is employed to simulate the scenario where a vehicle travels on uneven road surfaces. When a vehicle traverses such a road surface, the vehicle’s body acceleration undergoes periodic fluctuations, significantly impacting passenger comfort. Its mathematical expression is as follows:

(6) $$x_{R}=h\sin(\omega t)$$

where h stands for amplitude, ω represents angular frequency, and t represents time.

### 2.3. Controller Design

#### 2.3.1. PID Control

The PID controller is widely applied in numerous domains, including mechanical control, electronic device regulation, and industrial automation. It stands as a simple yet highly effective control methodology. Through the judicious tuning of PID parameters, it becomes feasible to improve system responsiveness, stability, and control accuracy. Presently, it boasts extensive utilization in the suspension systems of typical passenger automobiles.

Suspension control systems commonly utilize an incremental PID control algorithm, represented by the following equation:

(7) $$u(k)=u(k-1)+k_{P}[e(k)-e(k-1)]+k_{i}e(k)+k_{d}[e(k)-2e(k-1)+e(k-2)]$$

where u(k) represents the output value at the *k*-th sampling period, u(k−1) represents the output value at the (k−1)-th sampling period, e(k) denotes the input error at the *k*-th sampling period, e(k−1) denotes the input error at the (k−1)-th sampling period, $k_{P}$ represents the proportional coefficient, $k_{i}$ represents the integral coefficient, and $k_{d}$ represents the derivative coefficient.

#### 2.3.2. FNN-PID Control

**Figure 2:**
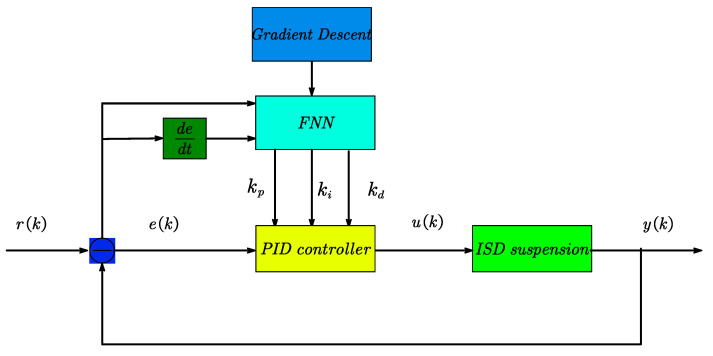
FNN-PID control.

The FNN-PID controller comprises a fuzzy neural network (FNN) and a conventional PID controller as shown in Figure 2 below. The fuzzy neural network (FNN) takes inputs including the spring mass acceleration error e(k) and the error rate of change $\frac{de}{dt}$. On the other hand, the PID controller takes only e(k) as input, while u(k) represents the control output. The system’s desired value is denoted as r(k), and the actual output is represented by y(k). After training with the fuzzy neural network algorithm, the optimal control parameters for the PID controller can be obtained. Based on these optimal control parameters, the PID controller adjusts the magnitude of the control output u(k) to achieve real-time control of the suspension system.

##### FNN-PID Algorithm

**Figure 3:**
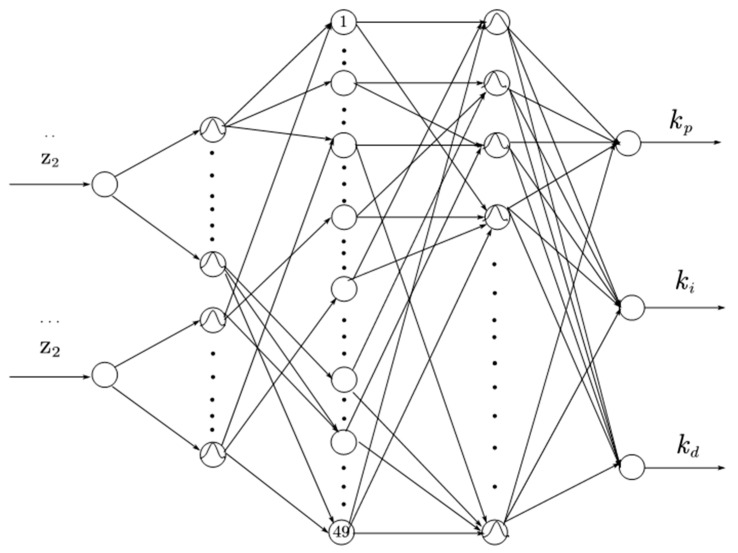
Fuzzy neural network’s structure.

Figure 3 shows the fuzzy neural network’s structure, which is a five-layer feed forward network with two inputs and three outputs.

Input layer: This layer has two nodes representing the acceleration error of the sprung mass and the rate of change in its acceleration error. The input and output expressions are:

(8) $$Out_{ij}^{1}=x_{i}$$

where $x_{i}$ represents the ith node.

Fuzzification layer: each input node of this layer corresponds to seven linguistic variable values, respectively, and since there are 2 nodes in the input layer, there are 14 nodes, and the input quantities are converted into fuzzy quantities after fuzzification in this layer, and then the affiliation function of each linguistic variable value is generated. The input and output expressions below:

(9) $$\left\{ \begin{array}{l} In_{ij}^{2}=Out_{ij}^{1}\\ Out_{ij}^{2}=\exp(-{\left(\frac{x_{i}\;-\;c_{ij}}{b_{ij}}\right)}^{2})\\ L_{i}^{j}(x_{i})=Out_{ij}^{2} \end{array}\right.$$

where $c_{ij}$ represents the centre of the affiliation function, $b_{ij}$ represents the width of the affiliation function, and $L_{i}^{j}(x_{i})$ represents the jth linguistic variable value of the *i*th node.

Fuzzy rule layer: Each node in this layer corresponds to a fuzzy rule, so there are 49 nodes in this layer. The nodes in this layer adopt the fuzziness of the error and the rate of change of the error in the fuzzification layer and use this as a preconditioning criterion for fuzzy inference output. The input–output expression is shown below:

(10) $$\left\{ \begin{array}{l} In_{i}^{3}=Out_{ij}^{2}\\ Out_{s}^{3}=L_{1}^{m}(x_{1})\cdot L_{2}^{n}(x_{2}) \end{array}\right.$$

where m = 1, 2, 3, 4, 5, 6, 7; n = 1, 2, 3, 4, 5, 6, 7; s is the rule node; and s = 1, 2, 3, ..., 49.

Normalization layer: this layer is the same as the fuzzy rule layer with 49 nodes, and its role is to calculate the normalization of the output of the fuzzy inference layer, with the input and output expressions as shown below:

(11) $$\left\{ \begin{array}{l} In_{s}^{4}=Out_{s}^{3}\\ Out_{s}^{4}=\frac{Out_{s}^{3}}{\sum_{i=1}^{49}Out_{i}^{3}} \end{array}\right.$$

where s = 1, 2, 3, ..., 49.

Output layer: the role of this layer is to convert the fuzzified values into the clarified values. There are three output nodes, which are $k_{P},k_{i},k_{d}$. The input and output expressions are shown below:

(12) $$\left\{ \begin{array}{l} In_{i}^{5}=Out_{s}^{4}\\ Out_{a}^{5}=\sum_{j=1}^{m}\omega_{ij}Out_{s}^{4} \end{array}\right.$$

where a = 1, 2, 3.

The final output is as follows:

(13) $$\left\{ \begin{array}{l} k_{p}=Out_{1}^{5}\\ k_{i}=Out_{2}^{5}\\ k_{d}=Out_{3}^{5} \end{array}\right.$$

Finally, the FNN-PID needs to be parameter optimized, given that the desired output value of the system is r(k), the actual output value is y(k), and the error function is shown in Equation (14) below:

(14) $$e=\frac{1}{2}{\left[r(k)-y(k)\right]}^{2}$$

Using the gradient descent method to learn the parameter updates for $c_{ij}$, $b_{ij}$ and $\omega_{ij}$. The learning algorithm is as follows in Equations (15)–(17).

(15) $$c_{ij}(k+1)=c_{ij}(k)-\eta \frac{\partial e}{\partial c_{ij}}+\alpha (c_{ij}(k-1)-c_{ij}(k-2))$$

(16) $$b_{ij}(k+1)=b_{ij}(k)-\eta \frac{\partial e}{\partial b_{ij}}+\alpha (b_{ij}(k-1)-b_{ij}(k-2))$$

(17) $$\omega_{ij}(k+1)=\omega_{ij}(k)-\eta \frac{\partial e}{\partial \omega_{ij}}+\alpha (\omega_{ij}(k-1)-\omega_{ij}(k-2))$$

#### 2.3.3. Optimal Fuzzy Neural Network PID Control Based on Grey Wolf Algorithm

**Figure 4:**
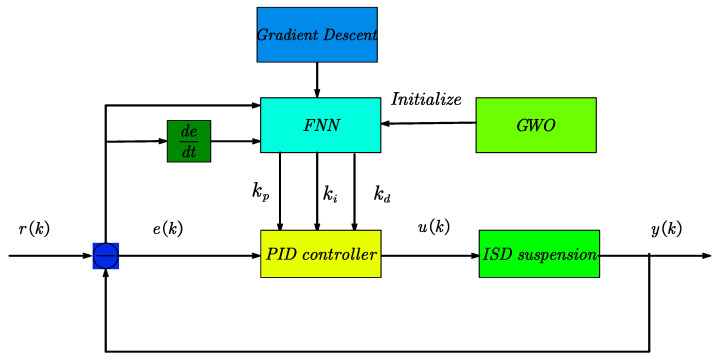
GWO-FNN-PID control structure.

On the basis of fuzzy neural network PID control, the optimization of the fuzzy neural network using grey wolf optimization (GWO) is proposed. Contrapose the characteristics that the initial parameters of the connection layer of the fuzzy neural network have a large influence on the effect of PID parameter tuning, the grey wolf optimization algorithm is used to search for the optimal initial parameters of the fuzzy neural network so that the fuzzy neural network obtains the optimal initial weights and clustering centres and widths, and then the fuzzy neural network with the optimal parameters is used for the tuning of the PID parameters, so as to achieve the optimal control effect. This controller combines the grey wolf’s ability to find the optimal and the fuzzy neural network’s ability to adaptively adjust, and has a strong tuning effect on the PID parameters. The structure of GWO-FNN-PID control is shown in Figure 4 below.

##### Grey Wolf Optimization Algorithm

**Figure 5:**
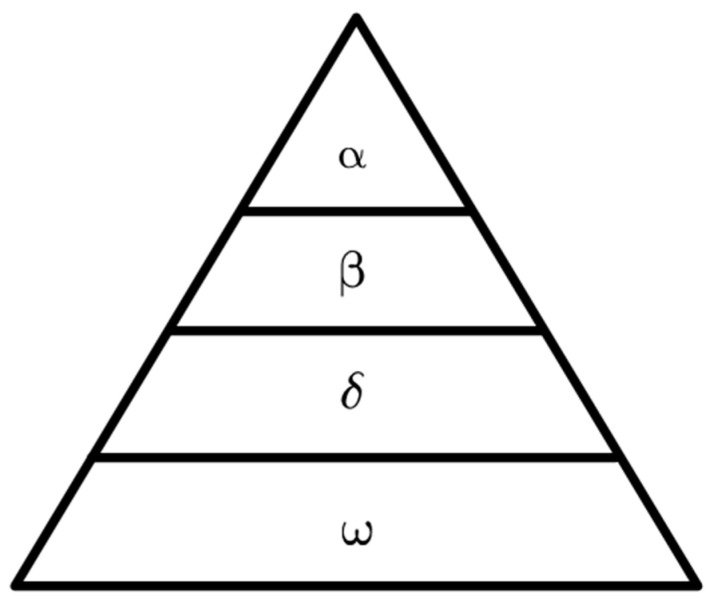
Grey wolf hierarchy.

In 2014, Mirjalili proposed the grey wolf optimization algorithm based on the predatory behaviour of grey wolves in nature. Its considers each wolf as a solution to the problem, the solution with the highest solution accuracy is called *α* wolf, the solution with the second highest solution accuracy is *β* wolf, the solution with the third highest solution accuracy is *δ* wolf, and the rest of the wolves are *ω* wolves. *ω* wolves are constantly updating their distance from their prey under the leadership of the *α*, *β*, and *δ* wolves, infinitely approaching their prey and capturing, tracking, rounding up, and attacking. The grey wolf hierarchy is shown below in Figure 5.

There are two main phases of prey encirclement:

1. Surrounding stage

When the location of the prey is determined, the wolf pack begins to pursue and encircle the prey, and the encirclement behaviour is represented by the following equation:

(18) $$d=|C\cdot x_{\omega p}(t)-x(t)|$$

where d represents the distance of the grey wolf population from the prey; $x_{\omega p}(t)$ represents the location of the prey; x(t) represents the location of the grey wolf population; C represents the system perturbation parameter; and t represents the number of iterations.

(19) $$\left\{ \begin{array}{l} A=2ar_{1}-a\\ a=2(1-\frac{t}{t_{\max}})\\ C=2r_{2} \end{array}\right.$$

where a represents convergence factor; and $r_{1},r_{2}$ are the random number.

A is a system parameter that varies within [−*a*, *a*]. When A ≥ 1, the wolf pack expands the search, i.e., corresponding to the global search, when A < 1, the wolf pack will contract the search scope, i.e., corresponding to the local optimization [32].

2. Hunting and attacking stage

When the wolf pack surrounds the prey in all directions, α-wolf, β-wolf, and δ-wolf will command ω-wolf to attack, the position of the prey will keep changing when it escapes, and ω-wolf can realize the dynamic movement of the encirclement circle, so as to maintain the all-round encirclement and attack of the prey, and ultimately to achieve the purpose of capturing the prey. Individual grey wolves update their position according to the following equation:

(20) $$\left\{ \begin{array}{l} d_{a}=|C_{1}\cdot x_{\alpha }(t)-x(t)|\\ d_{\beta }=|C_{2}\cdot x_{\beta }(t)-x(t)|\\ d_{\delta }=|C_{3}\cdot x_{\delta }(t)-x(t)| \end{array}\right.$$

where $d_{a}$ denotes the distance between the current wolf and the α wolf; $d_{\beta }$ denotes the distance between the current wolf and the β wolf; $d_{\delta }$ denotes the distance between the current wolf and the δ wolf; $C_{1}$ denotes the random perturbation of the α wolf; $C_{2}$ denotes the random perturbation of the β wolf; and $C_{3}$ denotes the random perturbation of the δ wolf:

(21) $$\left\{ \begin{array}{l} x_{1}=x_{\alpha }(t)-A_{1}\cdot d_{\alpha }\\ x_{2}=x_{\beta }(t)-A_{2}\cdot d_{\beta }\\ x_{3}=x_{\delta }(t)-A_{3}\cdot d_{\delta } \end{array}\right.$$

where $x_{1}$ represents the updated position of ω wolf after receiving α wolf command, $x_{2}$ represents the updated position of *ω* wolf after receiving *β* wolf command, $x_{3}$ represents the updated position of ω wolf after receiving δ wolf command, $A_{1}$ represents the random variable of α wolf, $A_{2}$ represents the random variable of β wolf, and $A_{3}$ represents the random variable of δ wolf.

The final position of the next generation wolf is shown in the following equation:

(22) $$x(t+1)=\frac{x_{1}+x_{2}+x_{3}}{3}$$

where x(t+1) denotes the position of the next generation *ω*.

##### Choice of the Fitness Function

The fitness function must accurately represent the quality of suspension smoothness and, as such, should consider three key performance indicators: vertical body acceleration (ACC), tire dynamic load (STD), and suspension working space (SWS). The fitness function is defined by the following equation:

(23) $$fitness=\frac{\mathrm{ACC}}{P_{1}}+\frac{\mathrm{STD}}{P_{2}}+\frac{\mathrm{SWS}}{P_{3}}$$

where $P_{1}$ represents vehicle body acceleration of passive suspension, $P_{2}$ represents tire dynamic load of passive suspension, $P_{3}$ represents suspension working space of passive suspension, ACC represents vehicle body acceleration of ISD suspension, STD represents tire dynamic load of ISD suspension, and SWS represents suspension working space of suspension.

##### Hybrid Algorithm Optimization Process

**Figure 6:**
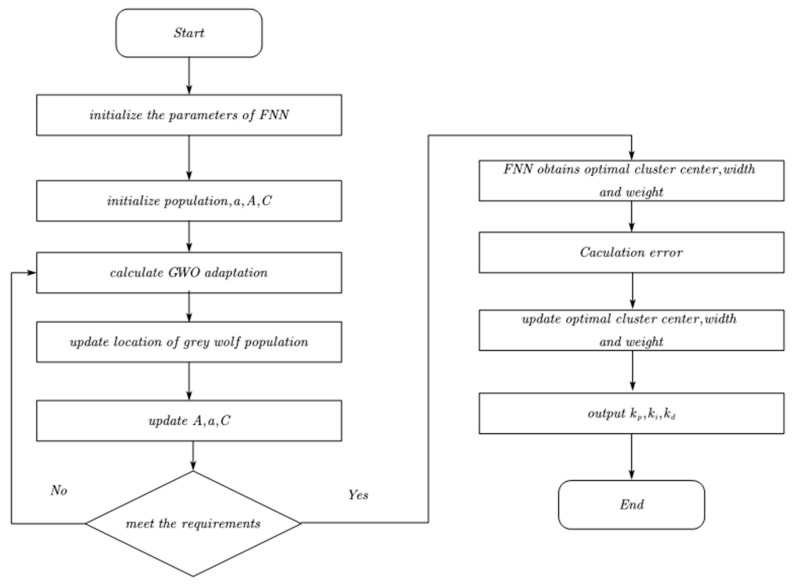
Flow chart of the GWO-FNN-PID algorithm.

The steps to optimize the fuzzy neural network using the grey wolf optimization algorithm are shown in Figure 6 below:

Step 1. Define the topology of fuzzy neural network and initialize the network’s parameters $\omega_{ij},b_{ij},c_{ij}$.

Step 2: Initialize the number of grey wolf populations and parameters a, A and C.

Step 3. Compute the fitness value for each wolf based on the equation 23 above, find the position of α wolf that is the optimal solution, the position of β wolf that is the suboptimal solution, and the position of δ wolf that is the third optimal solution, update the position of ω wolf according to the equation 22 above, and update the values of parameters a, A and C.

Step 4: Determine whether the maximum number of iterations is reached, if not, return to Step 3; if it is satisfied, determine the optimal parameters ($\omega_{ij},b_{ij},c_{ij}$) according to the current position of the α wolf, and then serve as the fuzzy neural network initial weights, affiliation centre, and width.

Step 5: Use gradient descent method to update the system parameters online.

Step 6: Final output $k_{P},k_{i},k_{d}$.

## 3. Simulation Analysis and Results

The ISD suspension system is simulated establishing Matlab/Simulink, and the 1/4 ISD suspension model and the random road excitation model are created in Simulink, respectively, and the basic parameters of the 1/4 ISD suspension are shown in Table 2 below.

**Figure 7:**
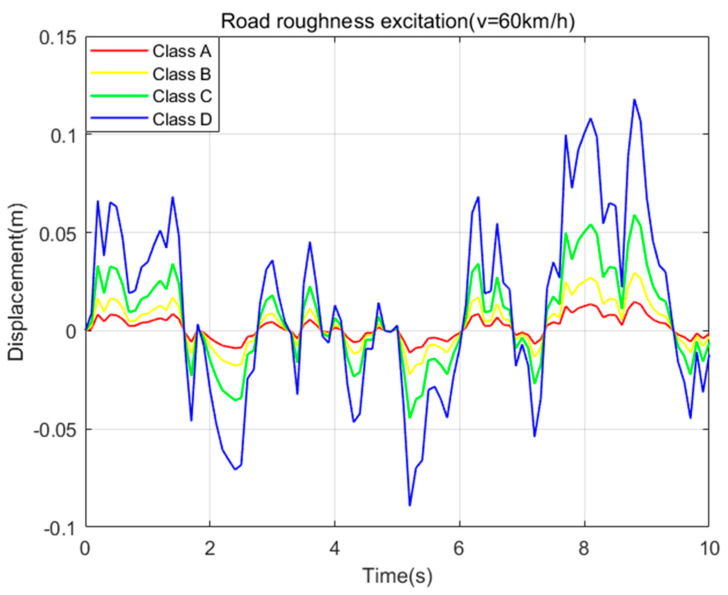
Road excitation time−domain curve (v = 60 km/h).

Using the filtered white noise of Equation (5) as the input signal of the road excitation, the values of *G*(*n*_0_) are selected as 16, 64, 256, and 1024, and the vehicle speed is selected as 60 km/h, and the time−domain curve of the road displacement of the road excitation model is shown in Figure 7 below.

**Figure 8:**
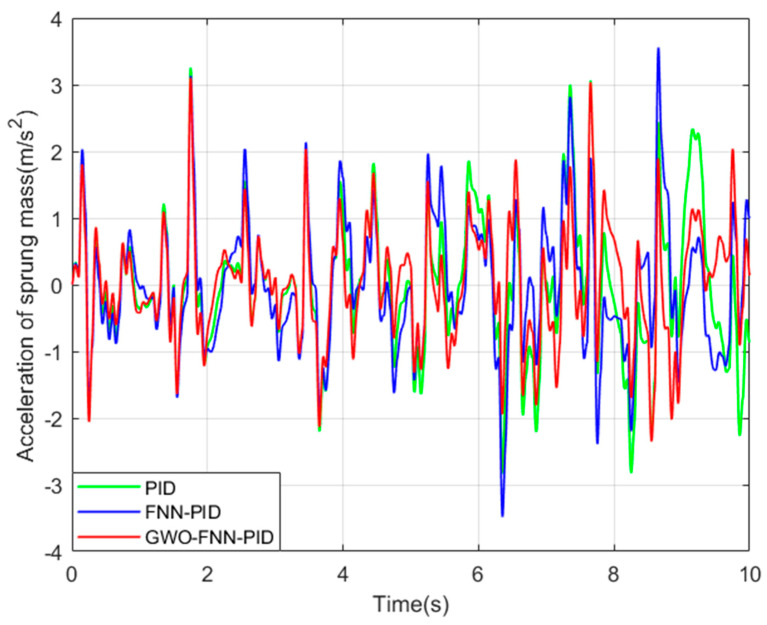
Acceleration of the sprung mass on a class C road.

**Figure 9:**
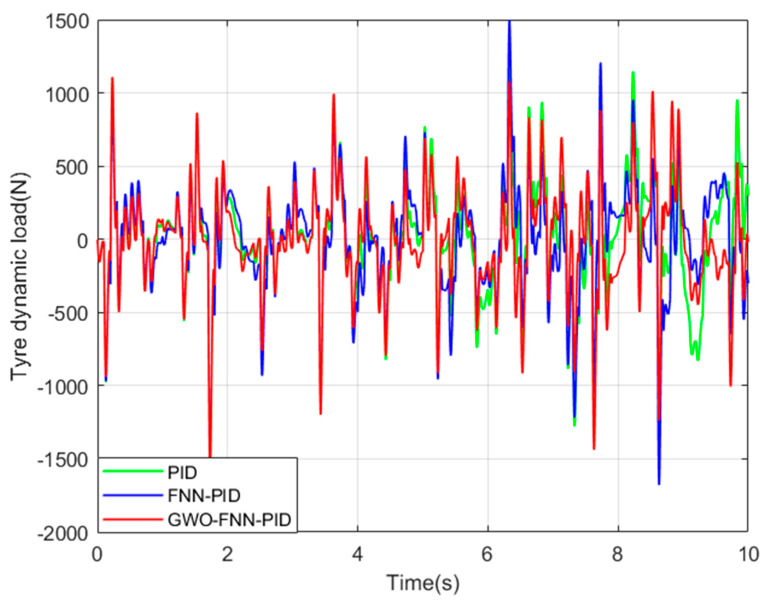
Tire dynamic load on a class C road.

**Figure 10:**
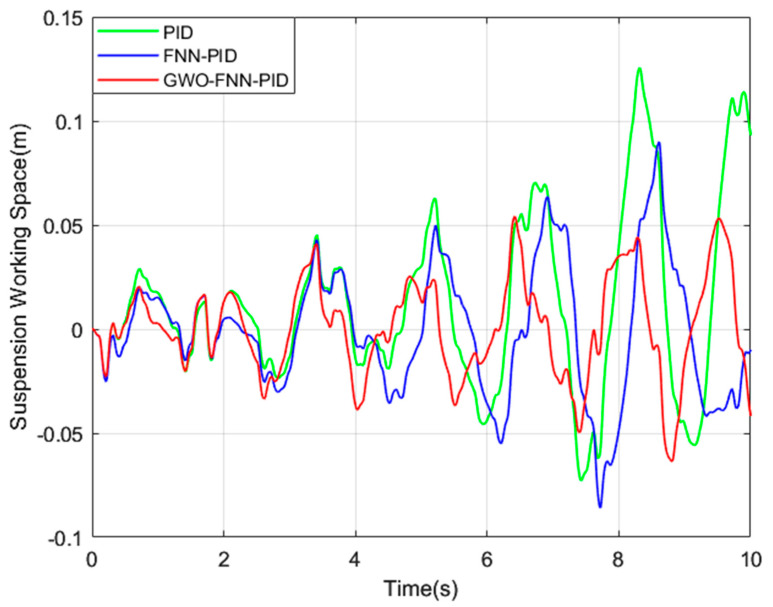
Suspension working space on a class C road.

To bolster the practicality of our simulation results and emphasize the exceptional performance of the GWO−FNN−PID control strategy, we performed simulations to assess the ISD suspension under PID, FNN−PID, and GWO−FNN−PID control strategies on road surfaces categorized as A, B, C, and D. These simulations were conducted over a 10 s interval, and the three parameters of the PID controller are set as follows: $k_{P}$ was 15, $k_{i}$ was 200, and $k_{d}$ was 0.02. The fuzzy neural network structure was set to 2−14−49−49−3, with a learning rate of 0.4. The grey wolf population size was set to 30, and the algorithm iteration count was 500, with a convergence factor of 2, and the resulting figures (Figure 8, Figure 9 and Figure 10) depict the spring mass acceleration (ACC), tire dynamic load (STD), and suspension working space (SWS) of the ISD suspension under class C road.

To provide a clearer analysis of the vibration damping performance of the ISD suspension with three control strategies, we present the root mean square values of the suspension’s output performance indicators under four levels of road excitation in Table 3 and Table 4.

Compared to the PID control strategy, the GWO−FNN−PID control strategy demonstrates notable reductions in the root mean square values of the spring-loaded mass acceleration by 18.4%, 17.7%, 17.4%, and 16.6%, the root mean square values of the tire dynamic load by 7.5%, 7.2%, 7.1%, and 6.7%, and the root mean square values of the suspension’s working space by 24.5%, 47.6%, 46.7%, and 45.5% for road types A, B, C, and D, respectively.

Comparatively, when compared to the FNN−PID control strategy, the GWO−FNN−PID control strategy exhibits reductions in the root mean square values of the sprung mass acceleration by 13.5%, 13.5%, 13.5%, and 13.5%, the root mean square values of the tire dynamic load by 3.2%, 3.2%, 3.1%, and 3.1%, and the root mean square values of the suspension’s working space by 19.2%, 26.3%, 26.3%, and 26.3%, respectively.

These results highlight the GWO−FNN−PID control strategy’s superior capability in reducing vehicle body acceleration and tire dynamic load compared to the PID and FNN−PID control strategies. As a result, it significantly enhances vehicle smoothness and driving safety.

**Figure 11:**
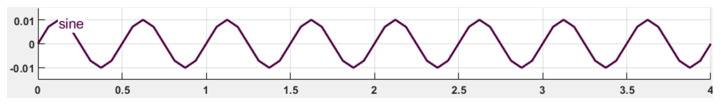
Sinusoidal road curve.

**Figure 12:**
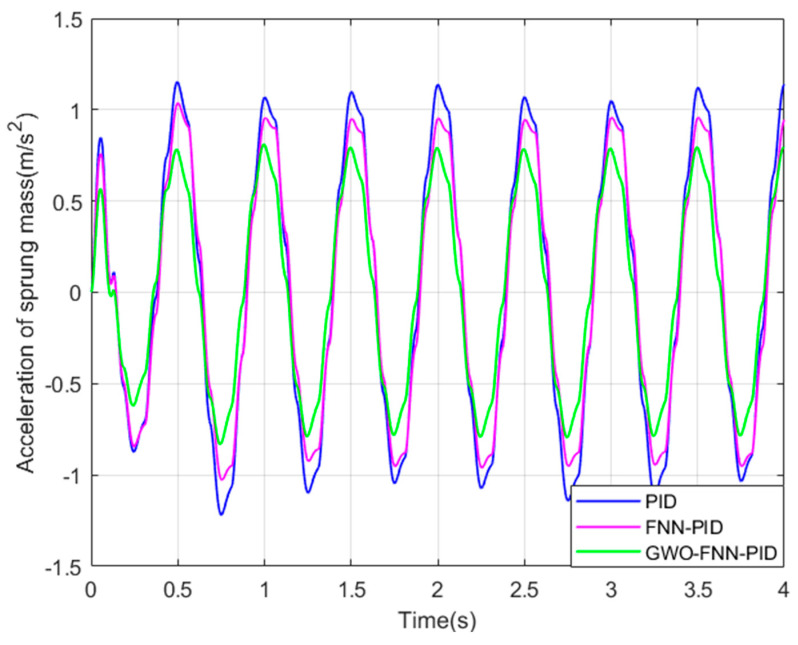
Acceleration of sprung mass on a sinusoidal road.

**Figure 13:**
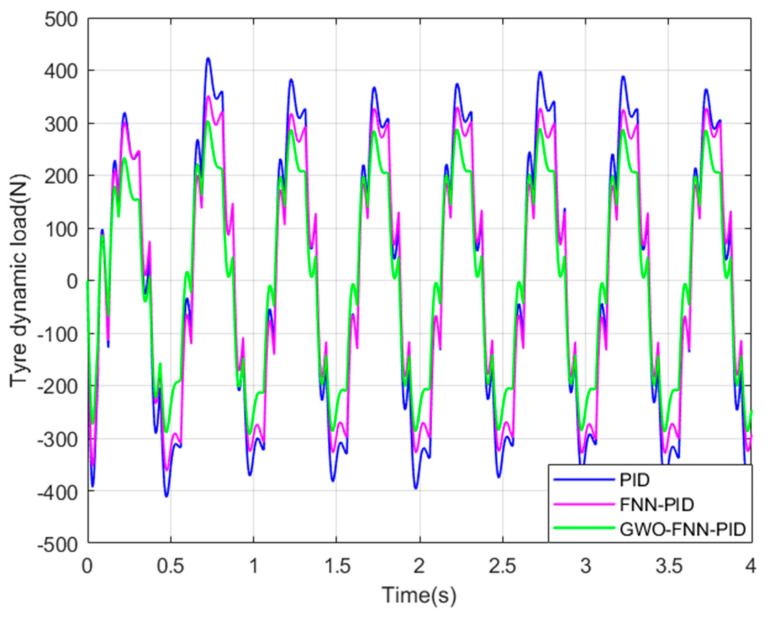
Tire dynamic load on a sinusoidal road.

**Figure 14:**
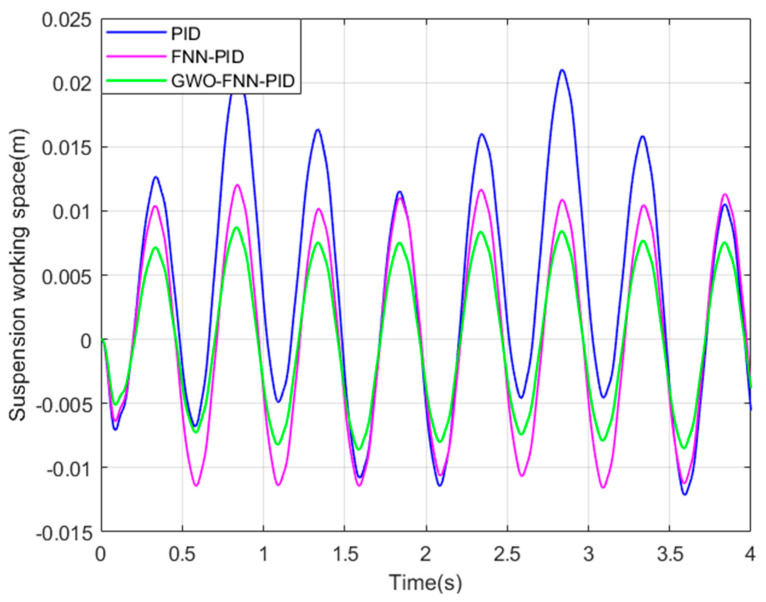
Suspension working space on a sinusoidal road.

In order to further validate the optimization effectiveness of GWO−FNN−PID, a sinusoidal excitation road profile was chosen as the input signal, as illustrated in Figure 11. Subsequently, additional simulations were performed using Matlab/Simulink, and the suspension performance indicators are displayed in Figure 12, Figure 13 and Figure 14.

The changes in suspension performance indicators under sinusoidal road surface signals can be clearly observed from Figure 12, Figure 13 and Figure 14. Compared to PID control, both FNN−PID control and GWO−FNN−PID control improved the ride comfort of the ISD suspension system. However, the damping effect of GWO−FNN−PID control is notably more pronounced.

Table 5 below presents the root mean square values of suspension performance indicators under sinusoidal road conditions. When compared to PID control and FNN−PID control, GWO−FNN−PID control resulted in a respective reduction of 29.8% and 21.2% in vertical body acceleration and a reduction of 12.2% and 17.8% in tire dynamic load. In terms of suspension working space, reductions of 67.6% and 28.7% were observed, respectively.

The simulation results unequivocally demonstrate that the damping effect achieved by the suspension system under the GWO−FNN−PID control strategy surpasses that of the same suspension system when subjected to PID control and FNN−PID control. As a result, the FNN−PID controller, optimized through the prowess of the grey wolf algorithm, exhibits significantly superior performance, offering substantial enhancements in both ride comfort and handling stability.

## 4. Conclusions

A 1/4 2-degree-of-freedom two-stage inerter–spring–damper (ISD) suspension simulation model was meticulously crafted using MATLAB/Simulink. Subsequently, a sophisticated GWO−FNN−PID control strategy was intricately devised based on this model. To fine-tune the initial parameters of the fuzzy neural network, a judicious blend of the grey wolf optimization algorithm and the gradient descent method was employed. This unique amalgamation of techniques mitigates the risk of becoming ensnared in local minima during the later stages of the learning process.

The simulation results unequivocally demonstrate that the damping effect achieved by the suspension system under the GWO−FNN−PID control strategy surpasses that of the same suspension system when subjected to PID control and FNN−PID control. As a result, the FNN−PID controller, optimized through the prowess of the grey wolf algorithm, exhibits significantly superior performance, offering substantial enhancements in both ride comfort and handling stability.

At present, our focus is on exploring the integration of the ISD semi−active suspension control system into the vehicle’s overall suspension system. We are diligently working towards achieving hardware–software integration to optimize cost control and enhance performance. In the future, we will continue to enhance the controller’s effectiveness, including the possibility of incorporating ADRC control algorithms.

## Figures and Tables

**Table 1:** Power spectral density of A–D road grade.

Road Grade	$G(n_{0})$
A	16
B	64
C	256
D	1024

**Table 2:** ISD suspension parameters.

Parameters	Units	Value
*m*_1_	kg	50
*m*_2_	kg	320
*c*_1_	N⋅s/m	1000
*c*_2_	N⋅s/m	1500
*k_t_*	N/m	190,000
*k*_1_	N/m	15,000
*k*_2_	N/m	20,000
*b*_1_	kg	500

**Table 3:** Root mean square value of suspension performance indexes for road A and B.

Index	Class A			Class B		
	ACC	STD	SWS	ACC	STD	SWS
PID	0.2563	103.4255	0.0106	0.507	205.9497	0.0225
FNN−PID	0.2415	98.7322	0.0099	0.4827	197.3164	0.016
GWO−FNN−PID	0.2089	95.6754	0.008	0.4175	191.2076	0.0118

**Table 4:** Root mean square values of suspension performance indexes for road C and D.

Index	Class C			Class D		
	ACC	STD	SWS	ACC	STD	SWS
PID	1.011	411.5452	0.044	2.0026	819.2036	0.0864
FNN−PID	0.9653	394.5796	0.0319	1.9299	788.8753	0.0639
GWO−FNN−PID	0.835	382.2369	0.0235	1.6692	764.0836	0.0471

**Table 5:** Root mean square values of suspension performance indexes for a sinusoidal road.

Controller	Performance		
	ACC	STD	SWS
PID	0.7397	244.8036	0.017
FNN−PID	0.6583	214.8947	0.0077
GWO−FNN−PID	0.5189	176.6557	0.0055

## Data Availability

Not applicable.
